# Hepatic steatosis, metabolic dysfunction and risk of mortality: findings from a multinational prospective cohort study

**DOI:** 10.1186/s12916-024-03366-3

**Published:** 2024-06-03

**Authors:** Ana-Lucia Mayén, Mirna Sabra, Elom K. Aglago, Gabriel Perlemuter, Cosmin Voican, Ines Ramos, Charlotte Debras, Jessica Blanco, Vivian Viallon, Pietro Ferrari, Anja Olsen, Anne Tjønneland, Fie Langmann, Christina C. Dahm, Joseph Rothwell, Nasser Laouali, Chloé Marques, Matthias B. Schulze, Verena Katzke, Rudolf Kaaks, Domenico Palli, Alessandra Macciotta, Salvatore Panico, Rosario Tumino, Claudia Agnoli, Marta Farràs, Esther Molina-Montes, Pilar Amiano, María-Dolores Chirlaque, Jesús Castilla, Mårten Werner, Stina Bodén, Alicia K. Heath, Kostas Tsilidis, Dagfinn Aune, Elisabete Weiderpass, Heinz Freisling, Marc J. Gunter, Mazda Jenab

**Affiliations:** 1 Nutrition and Metabolism Branch, International Agency for Research On Cancer (IARC-WHO), 25 Avenue Tony Garnier, Lyon, 69007 France; 2 Department of Epidemiology and Biostatistics, School of Public Health, Imperial College London, London, UK; 3 INSERM U996, Intestinal Microbiota, Macrophages and Liver Inflammation, DHU HepatinovLabex LERMIT, Clamart, France; 4 Faculté de Médecine Paris-Sud, Univ Paris-Sud, Université Paris-Saclay, Le Kremlin-Bicêtre, France; 5 Service d’hépato-Gastroentérologie, Hôpital Antoine-Béclère, Hôpitaux Universitaires Paris-Sud, Assistance Publique-Hôpitaux de Paris, Clamart, France; 6 Danish Cancer Society Research Center, Diet, Cancer and Health, Strandboulevarden 49, Copenhagen, 2100 Denmark; 7 Department of Public Health, Aarhus University, Bartholins Alle 2, Aarhus C, 8000 Denmark; 8 Université Paris-Saclay, UVSQ, Inserm “Exposome and Heredity” Team, CESP U1018, Gustave Roussy, Villejuif, France; 9 Dept. of Molecular Epidemiology, German Institute of Human Nutrition, Arthur-Scheunert-Allee 114-116, Nuthetal, 14558 Germany; 10 Department of Cancer Epidemiology, German Cancer Research Center (DKFZ), Heidelberg, Germany; 11 Cancer Risk Factors and Life-Style Epidemiology Unit, Institute for Cancer Research, Prevention and Clinical Network (ISPRO), Florence, Italy; 12 Department of Clinical and Biological Sciences, Centre for Biostatistics, Epidemiology, and Public Health (C-BEPH), University of Turin, Turin, Italy; 13 Dipartimento Di Medicina Clinica E Chirurgia, Federico II University, Naples, Italy; 14 Hyblean Association for Epidemiological Research, AIRE-ONLUS Ragusa, Ragusa, Italy; 15 Department of Research Epidemiology and Prevention Unit, Fondazione IRCCS Istituto Nazionale Dei Tumori, Milan, 20133 Italy; 16 Unit of Nutrition and Cancer, Epidemiology Research Program, Catalan Institute of Oncology (ICO), Bellvitge Biomedical Research Institute (IDIBELL), Barcelona, L’Hospitalet de Llobregat 08908 Spain; 17 Department of Nutrition and Food Science, Campus of Cartuja, University of Granada, Granada, 18071 Spain; 18 Spanish Consortium for Research On Epidemiology and Public Health (CIBERESP), Instituto de Salud Carlos III, Madrid, 28029 Spain; 19 Instituto de Investigación Biosanitaria Ibs. Granada, Granada, 18012 Spain; 20 Institute of Nutrition and Food Technology (INYTA) ‘José Mataix’, Biomedical Research Centre, University of Granada, Granada, 18071 Spain; 21 Ministry of Health of the Basque Government, Sub Directorate for Public Health and Addictions of Gipuzkoa, San Sebastian, Spain; 22 Biodonostia Health Research Institute, Epidemiology of Chronic and Communicable Diseases Group, San Sebastián, Spain; 23 Department of Epidemiology, Murcia Regional Health Council, IMIB-Arrixaca,, Murcia University, Murcia, Spain; 24 Navarra Public Health Institute – IdiSNA, Pamplona, Spain; 25 Department of Public Health and Clinical Medicine, Medicine, Umeå University, Umeå, SE-901 87 Sweden; 26 Department of Clinical Sciences, Pediatrics, Umeå University, Umeå, SE-901 87 Sweden; 27 Department of Nutrition, Oslo New University College, Oslo, Norway; 28 Department of Endocrinology, Morbid Obesity and Preventive Medicine, Oslo University Hospital Ullevål, Oslo, Norway; 29 Office of the Director, International Agency for Research On Cancer (IARC-WHO), Lyon, France

**Keywords:** Hepatic steatosis, Metabolic syndrome, Mortality, Phenotypic NASH, MAFLD

## Abstract

**Background:**

Non-alcoholic fatty liver disease (NAFLD) and metabolic syndrome (MetS) are implicated in the aetiology of non-communicable diseases. Our study aimed to evaluate associations between NAFLD and MetS with overall and cause-specific mortality.

**Methods:**

We used dietary, lifestyle, anthropometric and metabolic biomarker data from a random subsample of 15,784 EPIC cohort participants. NAFLD was assessed using the fatty liver index (FLI) and MetS using the revised definition. Indices for metabolic dysfunction–associated fatty liver disease (MAFLD) were calculated. The individual associations of these indices with overall and cause-specific mortality were assessed using multivariable Cox proportional hazards models to estimate hazard ratios (HRs) and 95% confidence intervals (95%CIs). As a subobjective, risk associations with adaptations of new classifications of metabolic dysfunction–associated steatotic liver disease (MASLD) and metabolic and alcohol-related liver disease (MetALD) were also assessed.

**Results:**

Among the 15,784 sub-cohort participants, a total of 1997 deaths occurred (835 due to cancer, 520 to CVD, 642 to other causes) over a median 15.6 (IQR, 12.3–17.1) years of follow-up. Compared to an FLI < 30, FLI ≥ 60 was associated with increased risks of overall mortality (HR = 1.44, 95%CI = 1.27–1.63), and deaths from cancer (HR = 1.32, 95%CI = 1.09–1.60), CVD (HR = 2.06, 95% CI = 1.61–2.63) or other causes (HR = 1.21, 95%CI = 0.97–1.51). Mortality risk associations were also elevated for individuals with MAFLD compared to those without. Individuals with MetS were at increased risk of all mortality endpoints, except cancer-specific mortality. MASLD and MetALD were associated with higher risk of overall mortality.

**Conclusions:**

Our findings based on a prospective cohort suggest that individuals with hepatic steatosis or metabolic dysfunction have a higher overall and cause-specific mortality risk.

**Supplementary Information:**

The online version contains supplementary material available at 10.1186/s12916-024-03366-3.

## Background

Non-alcoholic fatty liver disease (NAFLD) is a chronic disease affecting approximately 25% of adults globally [1]. It is defined as an accumulation of fat in the liver not due to excess alcohol intake [2] and can range from simple steatosis to a severe form known as non-alcoholic steatohepatitis (NASH) [3, 4]. Major risk factors for NAFLD include unhealthy dietary and lifestyle habits [5]. Definition and classification of NAFLD was updated in 2023 as metabolic dysfunction–associated steatotic liver disease (MASLD), with the term metabolic and alcohol-related liver disease (MetALD) describing a subset of MASLD in conjunction with higher alcohol consumption levels [6].

The gold standard method for clinical diagnosis of NAFLD is liver biopsy, although in clinical practice radiological tests are more frequently performed [7]. Liver biopsy is invasive, costly and not easily implemented in large cohorts or population-based research settings. Several non-invasive and less-expensive methods for diagnosing hepatic steatosis are based on anthropometry measures and levels of circulating metabolic biomarkers [7]. These include the fatty liver index (FLI) [8] and the metabolic dysfunction–associated fatty liver disease (MAFLD) index [9]. Calculation of such scores may be more feasible for population-wide risk stratification.

NAFLD can cause liver damage and increase the risk of hepatocellular cancer (HCC) [10]. It is also associated with increased risk of cardiovascular diseases (CVD), diabetes and many cancers, increasing the risk of mortality [11]. Current evidence on the association of NAFLD with mortality comes mostly from studies based on imaging or liver biopsy diagnoses within population- or hospital-based settings. Two earlier meta-analyses did not demonstrate any associations with all-cause mortality [1, 12], but a more recent meta-analysis showed a positive risk association [13]. There is little evidence from prospective cohort studies using non-invasive indices of NAFLD evaluating its risk associations with all-cause and cause-specific mortality.

NAFLD is often described as the hepatic expression of the metabolic syndrome (MetS), a cluster of metabolic disorders including central obesity, systemic hypertension, insulin resistance and dyslipidaemia [14]. Individuals with MetS have a higher risk of chronic disease development [15, 16], and data from prospective cohorts report positive associations between MetS and risk of all-cause and CVD mortality [17–22], but evidence evaluating associations with cancer mortality is scarce. It is still unclear whether any impact of MetS pertains to the cluster of metabolic abnormalities together or to any individual component [23].

In this study, using data from a subset of participants from the multinational European Prospective Investigation into Cancer and Nutrition (EPIC) cohort, we investigated associations between NAFLD (using FLI), MAFLD and MetS with the risk of all-cause and cause-specific mortality.

## Methods

### Study population

EPIC is a prospective cohort study of more than 521,324 participants aimed to assess cancer and chronic disease risk factors. The study design has been previously described [24, 25]. Briefly, participants were recruited between 1992 and 2000. Blood samples were collected from 385,747 of the 519,978 EPIC study participants and 346,055 of 455,680 individuals participating in eight out of ten EPIC countries (Denmark, France, Germany, Italy, the Netherlands, Spain, Sweden and UK) that also participated in the InterAct sub-study nested within EPIC [26], used as a basis for the present analysis. Individuals without available blood samples (*n* = 109,625) or without information on reported diabetes status (*n* = 5821) were excluded, leaving a total of 340,234 participants eligible for inclusion in the InterAct sub-study [26]. A representative random sub-cohort stratified by centre was selected among these eligible participants, constituting a case-cohort design. In the present analyses, we excluded the Umeå centre of Sweden due to missing values for waist circumference (WC) (*n* = 1050). For the present analysis, we relied on a subset of 15,784 EPIC participants, for whom data to calculate the various FLI, MAFLD or MetS risk scores were available. As a subobjective, we also assessed risk associations for adaptations of MASLD and MetALD (calculated indices based on available data within our cohort and using FLI to identify hepatic steatosis) which have been adopted as replacement definitions for NAFLD. As a further subobjective, we also assessed the phenotypically defined NASH score [8], as a non-invasive but incompletely validated scoring index for NASH. Each participant provided lifestyle and dietary information at baseline using validated, dietary and lifestyle questionnaires. Anthropometric measures were assessed at the baseline examination by trained clinical staff.

### Assessment of NAFLD

The FLI, a validated algorithm of NAFLD developed by Bedogni et al. [8], was estimated using the following formula:

$$FLI=\frac{(e(0.953\times\ln(TG)+0.139\times BMI+0.718\times\ln(GGT)+0.053\times WC-15.745))}{(1+e(0.953\times\ln(TG)+0.139\times BMI+0.718\times\ln(GGT)+0.053\times WC-15.745}X\;100$$

where TG stands for triglycerides, BMI for body mass index, GGT for Gamma-Glutamyl-Transferase, and WC for waist circumference. The units of TG, GGT and WC were mg/dL, U/L and cm, respectively. An FLI ≥ 60 indicates presence of NAFLD whereas an FLI < 30 rules out NAFLD [8]. To further investigate the presence of NAFLD in individuals with a stronger presence of risk factors (e.g., higher BMI, WC), we also assessed risk associations with an unvalidated category of FLI ≥ 80 which we speculate may indicate more severe NAFLD.

MAFLD is defined as liver steatosis together with metabolic dysfunction [9]. MAFLD consists of overweight (BMI ≥ 25 kg/m^2^), type 2 diabetes mellitus (antidiabetic drug use or fasting plasma glucose ≥ 7.0 mmol/L), or a combination of at least two of the following: (i) WC ≥ 102 cm for men and ≥ 88 cm for women, (ii) blood pressure (BP) ≥ 130/85 mmHg or antihypertensive drug use, (iii) plasma TG ≥ 1.70 mmol/L or lipid-lowering drug treatment, (iv) high-density lipoprotein (HDL) cholesterol < 1.0 mmol/L for men and < 1.3 mmol/L for women or lipid-lowering drug treatment, (v) prediabetes defined as fasting plasma glucose of 5.6–6.9 mmol/L, (vi) C-reactive protein (hs-CRP) > 2 mg/L, (vi) homeostatic model assessment of insulin resistance (HOMA-IR) ≥ 2.5. In our data, HOMA-IR, medication use and some other data were unavailable. Thus, we modified the original formula [27]: (a) we utilized self-reported diabetes information collected at baseline, (b) we used baseline BP measures taken by trained personnel without consideration of antihypertensive medication use, (c) plasma glucose measures were applied without consideration of fasting status. Almost half of participants (46%) were not fasting when blood samples were collected.

### Assessment of calculated indices of MASLD and MetALD

We also used the new classification proposed to re-define NAFLD, defined as MASLD. We adapted the original formula for these classifications [6] to reflect the data availability and structure within our cohort with the following definitions specific to this analysis. Participants with MASLD were defined as having an FLI ≥ 60 and at least one of the following: (i) BMI > 25 kg/m^2^ or WC > 94 cm in men or > 80 cm in women; (ii) (non)fasting glucose ≥ 5.6 mmol/L (100 mg/dL) or HbA1c ≥ 5.7% (39 mmol/L) or self-reported diabetes at baseline; (iii) elevated BP (systolic BP ≥ 130 mmHg, diastolic BP ≥ 85 mmHg); (iv) plasma triglycerides ≥ 1.70 mmol/L (150 mg/dL); (v) plasma HDL cholesterol ≤ 1.0 mmol/L (40 mg/dL) in men and ≤ 1.3 mmol/L (50 mg/dL) in women. We also classified individuals with MetALD as those with MASLD who consume greater amounts of alcohol per week (140–350 g/week and 210–420 g/week for females and males, respectively).

### Assessment of MetS

The presence of MetS was determined using the International Diabetes Federation (IDF) definition [28], where MetS is defined as: central obesity (WC > 94 cm in men or > 80 cm in women), plus any two of the following four factors: (i) raised TG (≥ 150 mg/dL or specific treatment for this lipid abnormality), (ii) reduced HDL cholesterol (< 40 mg/dL in men, < 50 mg/dL in women or specific treatment, (iii) raised BP (systolic BP ≥ 130 mmHg, diastolic BP ≥ 85 mmHg or treatment of previously diagnosed hypertension), (iv) raised fasting plasma glucose (FPG) ≥ 100 mg/dL or previously diagnosed type 2 diabetes. We modified the formula to include plasma glucose instead of fasting plasma glucose. MetS was calculated as follows [29]:

$$MetS\;score\;in\;men=\frac{\displaystyle\frac{waist}{height}}{0.5}+\frac{glucose}{5.6}+\frac{elevated\;triglycerides}{1.7}+\frac{systolic\;blood\;pressure\;\;}{130}-\frac{HDL\;cholesterol\;}{1.02}$$

$$MetS\;score\;in\;women=\frac{\displaystyle\frac{waist}{height}}{0.5}+\frac{glucose}{5.6}+\frac{elevated\;triglycerides\;}{1.7}+\frac{systolic\;blood\;pressure\;\;}{130}-\frac{cholesterol}{1.28}$$

where WC and height were measured in centimeters; glucose, TG and HDL in mmol/L, and systolic BP in mmHg. In our analyses, MetS as a continuous variable excludes those with FLI < 30.

We also modelled associations between MetS and all-cause and cause-specific mortality using the harmonized definition proposed by IDF [30] and the modified version of the National Cholesterol Education Program (NCEP) definition, for which we also included abdominal obesity (WC ≥ 102 cm in men and ≥ 88 cm in women) [31].

### Case ascertainment

Vital status, cause and date of death were ascertained using record linkage with cancer registries, boards of health, death registries, or by active follow-up. Data were coded using the 10th revision of the International Statistical Classification of Diseases, Injuries and Causes of Death (ICD-10) where the underlying cause was the official cause of death. Four different cause-specific deaths were selected: CVD (I00–I99 excluding I20–I25) and coronary heart disease (CHD) (I20–I25) (both described in this manuscript as CVD); cancer deaths (C00-C97 and B21); a group for all other causes (including external causes of morbidity and mortality (V01–Y98); unknown causes (R96–R99), digestive (K00-K95) and respiratory diseases (J00-J99)).

### Statistical analyses

Participant characteristics were described using mean ± standard deviation (SD) and percentages. FLI (FLI < 30, FLI ≥ 30 and < 60, FLI ≥ 60), MetS (yes, no), MAFLD (yes, no), MASLD (yes, no), MetALD (yes, no) and phenotypic NASH (yes, no) were modelled as categorical variables. FLI and MetS were also modelled as continuous variables. We used Cox proportional hazards regression models to estimate hazard ratios (HR) and 95% confidence intervals (CI) between each exposure and all-cause and cause-specific mortality. Entry time was considered as age at recruitment and exit time was either age at death or censoring date (lost to or end of follow‐up), whichever came first. For each index, we assessed 2 separate models. Model 1: stratified by age, study centre and sex. Model 2: additionally adjusted for smoking status (never, current, former), physical activity using the Cambridge index (inactive, moderately inactive, moderately active, active, missing), alcohol intake during lifetime (never, light or never heavy; heavy or light former; periodically or always heavy; missing), fasting status at blood collection (< 3 h since last meal (no), 3–6 h (in between), > 6 h (yes)), tertiles of Mediterranean diet score (range 0 to 9), HbA1c serum levels and highest level of education attainment (none, primary school, technical/professional, secondary, longer, missing). This confounder adjustment strategy considers major lifestyle factors or biochemical measures that may have modified the findings based on current knowledge. We assessed the associations between each individual component of the MetS (abnormal glucose metabolism, elevated TG, elevated BP, abdominal obesity, reduced HDL cholesterol and BMI per 1 kg/m^2^ unit increase) and the mortality risk using the same models. As a subobjective, we tested whether associations between each NAFLD or MetS index and mortality were modified by a priori defined covariates: sex, age in categories (< 45, 45–65, > 65 years), presence of diabetes, highest level of education attained, menopausal status (women only), level of alcohol consumption at baseline (sex-specific tertiles of alcohol intake), smoking status, physical activity and levels of hsCRP as a circulating marker of chronic inflammation. Assessments of interaction were obtained using a likelihood ratio test of models with and without the interaction term. Analyses were conducted using STATA, version 14. Missing values in covariates were imputed (TG, WC, systolic and diastolic BP) using multivariate imputation by chained equations (MICE) in SAS (SAS/STAT 15.1) with the PROC MI command.

### Sensitivity and additional analyses

In sensitivity analyses, we excluded deaths that occurred during the first 2 years of follow‐up to minimize reverse causation. We additionally adjusted model 2 for circulating hsCRP levels to rule out residual confounding due to inflammation, except for analyses focusing of MAFLD as the main exposure because hs/CRP is part of the formula to compute MAFLD. In additional models, we excluded participants in the highest sex-specific tertile of alcohol consumption at baseline or those participants who indicated being always heavy drinkers for alcohol intake during their lifetime (26 to 276 g/day of alcohol in men, 8 to 143 g/day of alcohol in women), participants who were periodically or always heavy drinkers, who reported a cardiovascular problem at baseline or who reported any type of incident cancer. We also calculated the mortality risk for participants with phenotypic NASH using an index measured as elevated alanine aminotransferase (ALT) (≥ 40 IU/L) and at least 3 out of the 5 following metabolic derangements [9]: (i) BMI ≥ 30 kg/m^2^ or WC ≥ 102 cm in men and ≥ 88 cm in women, (ii) blood glucose ≥ 110 mg/dL, HbA1c ≥ 5.7% or drug therapy for diabetes, (iii) HDL cholesterol ≤ 40 mg/dL in men and ≤ 50 mg/dL in women, (iv) TG ≥ 150 mg/dL while fasting or drug therapy for elevated TG, (v) BP ≥ 135/85 mmHg or drug therapy for hypertension. Having no data on diabetes or dyslipidemia medication use, we defined these variables based on self-reported diabetes at baseline. The phenotypically defined NASH score has not been fully validated [32, 33].

We also identified the overlap between indices by identifyingc:(1) FLI ≥ 60 and MAFLD; (2) FLI ≥ 60, MAFLD and MetS; (3) FLI ≥ 60, MAFLD and phenotypic NASH; (4) FLI ≥ 60, MAFLD, MetS and phenotypic NASH. Finally, we computed the Kaplan Meier curves for all-cause and cause-specific mortality by NAFLD and MetS.

## Results

Table 1 shows the baseline characteristics of the population. Individuals with NAFLD and MetS had a higher intake of daily calories and BMI, were more frequently physically inactive, periodically, or always heavy drinkers, and more frequently had diabetes. During a mean follow-up of 14.4 years, 1997 deaths were registered within the 15,784 participants. Of these deaths, 835 were due to cancer, 520 due to CVD and 642 due to any other cause of death.

**Table 1:** Baseline characteristics of participants by categories of FLI, MAFLD, phenotypic NASH and MetS categories, European Prospective Investigation into Cancer, 1992–2000 (*n* = 15,784)

**Characteristics**	**FLI**			**MAFLD**		**Phenotypic NASH**		**MetS IDF 2006**	
	**FLI < 30**	**FLI ≥ 30 and < 60**	**FLI ≥ 60**	**No**	**Yes**	**No NASH**	**NASH**	**No MetS**	**MetS**
Age, years, mean ± SD	51.5 ± 9.3	54.2 ± 8.5	54.8 ± 7.9	52.4 ± 9.1	54.8 ± 7.9	53.0 ± 8.9	52.9 ± 8.5	52.4 ± 9	54.9 ± 8.1
Women, %	81	48	36	70	36	64	33	64	57
Dietary variables, mean ± SD or %									
Total energy intake, kcal/day	2130 ± 582	2265 ± 646	2351 ± 697	2175 ± 608	2348 ± 697	2210 ± 630	2309 ± 682	2213 ± 627	2226 ± 657
Relative Mediterranean diet Score^a^									
Tertile 1	25	29	34	34	41	35	41	35	35
Tertile 2	46	43	41	35	31	34	33	35	33
Tertile 3	28	28	25	31	28	31	26	30	32
Alcohol drinking history, %									
Never, light or never heavy drinkers	67	62	59	65	59	64	57	64	61
Heavy or light former drinker	5	7	6	6	6	6	5	5	8
Periodically or always heavy drinker	8	12	18	10	18	11	15	11	13
Missing	20	19	18	19	18	19	23	19	18
BMI, kg/m^b^ ± SD	23.6 ± 2.6	27.2 ± 2.7	30.9 ± 4.1	24.8 ± 3.1	31.0 ± 4.0	26.1 ± 4.3	27.9 ± 4.1	25.3 ± 3.8	29.5 ± 4.0
Waist circumference, cm ± SD	77.6 ± 7.8	91.2 ± 6.7	102.0 ± 9.0	82.0 ± 9.8	102.2 ± 9.0	86.3 ± 12.7	94.7 ± 12.3	83.7 ± 11.9	97.0 ± 10.6
Physical activity (Cambridge index), %									
Inactive	21	26	28	22	28	24	25	22	31
Moderately inactive	35	32	33	34	32	34	34	33	34
Moderately active	23	20	20	22	20	22	20	23	18
Active	20	20	19	20	19	20	20	21	16
Missing	1	2	1	1	1	1	2	2	1
Smoking status, %									
Never	50	44	38	48	38	46	37	45	47
Former smoker	24	29	33	26	33	27	35	27	27
Current smoker	25	26	27	25	27	26	27	26	25
Missing	1	1	1	1	1	1	1	1	1
Education, %									
None	5	11	13	7	13	9	6	7	14
Primary completed	29	37	38	32	38	33	34	31	40
Technical/ professional	23	22	22	23	22	23	23	23	20
Secondary	17	11	9	15	9	14	11	15	10
Tertiary	23	18	16	21	15	20	24	22	14
Missing	2	2	2	2	2	2	2	2	1
HbA1c > 6.5%^b^	1	2	8	1	8	3	8	1	8
Glucose, median, (Q4)	4.8 (5.3)	5.1 (5.6)	5.3 (6.0)	4.9 (5.4)	5.4 (6.1)	5.0 (5.5)	5.4 (6.3)	4.9 (5.4)	5.3 (6.0)
Fasting status, %									
No	41	39	40	40	40	40	41	41	35
In between	15	17	19	16	19	16	19	17	16
Yes	30	31	27	31	27	30	23	28	35
Missing	14	13	13	14	13	14	17	14	14
hs-CRP, ng/ml, median, (Q4)	790.0 (1,627.8)	1,330.0 (2,640.0)	2000.0 (3,960.0)	940.0 (1940.0)	2,030 (4,000.0)	1,100.0 (2,380.0)	1,490.0 (3,010.0)	980.0 (2,075.0)	1770.0 (3,630.0)
HDL cholesterol, mmol/l, median (Q4)	1.6 (1.9)	1.3 (1.6)	1.2 (1.4)	1.5 (1.8)	1.2 (1.4)	1.5 (1.8)	1.2 (1.5)	1.5 (1.8)	1.2 (1.5)
Triglycerides, mmol/l, median (Q4)	0.9 (1.2)	1.3 (1.8)	1.9 (2.7)	1.0 (1.4)	1.9 (2.6)	1.1 (1.6)	1.8 (2.7)	1.3 (1.8)	1.9 (2.7)
ASAT, IU/l, median (Q4)	25.0 (29.0)	28.0 (32.0)	30.9 (37.0)	26.0 (30.0)	31.0 (37.0)	26.3 (31.0)	43.0 (52.0)	27.0 (31.0)	28.0 (33.0)
ALAT, IU/l, median (Q4)	16.0 (20.0)	20.0 (27.0)	26.0 (37.0)	17.0 (22.0)	25.9 (37.0)	18.0 (24.0)	50.0 (62.0)	18.0 (24.0)	22.0 (30.0)
GGT, mg/dL, median (Q4)	16.0 (21.0)	24.0 (35.0)	38.0 (62.0)	18.0 (26.0)	38.0 (61.0)	20.0 (30.0)	56.0 (97.0)	19.0 (31.0)	25.0 (41.0)
Elevated blood pressure^c^									
Yes, %	21	34	46	25	46	29	47	18	70

The distribution by country is defined as follows: France (4%), Italy (13%), Spain (24%), UK (9%), Netherlands (10%), Germany (14%), Sweden (12%) and Denmark (14%)

*ASAT* aspartate aminotransferase, *ALAT* alanine transaminase, *BMI* body mass index, *GGT* gamma-glutamyl transferase, *hs-CRP* high-sensitivity C-reactive protein, *FLI* fatty liver index, *HDL* high-density lipoprotein, *IDF* International Diabetes Federation, *MAFLD* metabolic dysfunction-associated fatty liver disease, *MetS* metabolic syndrome, *Q4* quartile 4

^a^rMED score is based on tertiles of Mediterranean diet score (ranged 0 to 9, considering the combined intake of fruits, vegetables, legumes, cereals, lipids, fish, dairy products, meat products and alcohol intake). Each rMED component (apart from alcohol) was measured as g per 2000 kcal/day (to express intake as energy density) and was divided into tertiles of dietary intake

^b^Diabetes defined according to the National Glycohemoglobin Standardization Program (HbA1c > 6.5%)

^c^Elevated blood pressure refers to ≥ 130/85 mmHg

### All-cause mortality

Table 2 shows associations between NAFLD and MetS indices with all-cause mortality. FLI was positively associated with the risk of all-cause mortality with a HR 1.12 (95%CI = 1.09–1.16) per 10-point increase. Participants with an FLI ≥ 60 demonstrated a HR of 1.44 (95%CI = 1.27–1.63) whereas those with the arbitrary cut-point of FLI ≥ 80 showed a HR of 1.74 (95%CI = 1.50–2.01) for all-cause mortality, as compared to participants with FLI < 30 (Additional file 1: Supplementary Table 1). MetS was positively associated with risk of all-cause mortality, with a HR of 1.42 (95%CI = 1.30–1.54) per 1-unit increase, and 37% higher risk of all-cause mortality in those with MetS (HR 1.37, 95%CI = 1.24–1.52), compared to those without MetS. Having MAFLD was positively associated with all-cause mortality with HRs of 1.39 (95% CI = 1.25–1.55). Associations for all-cause mortality followed the same pattern by sex. We found evidence for heterogeneity of associations by alcohol intake at baseline for FLI (Additional file 1: Supplementary Table 2) with a p-interaction = 0.003 with the strongest association seen in heavy drinkers with FLI ≥ 60 compared to light drinkers with FLI < 30. As for MetS components, abnormal glucose metabolism appeared to be the strongest driver for the observed positive association of MetS with all-cause mortality (Additional file 1: Supplementary Table 3).

**Table 2:** HRs (95% CIs) for all-cause mortality according to FLI, MAFLD and MetS indices, European Prospective Investigation into Cancer, 1992–2000 (*n* = 15,784)

	**FLI**				**MAFLD**		**MetS IDF 2006**		
	**FLI < 30**	**FLI ≥ 30 and < 60**	**FLI ≥ 60**	**FLI (per each 10-point increase)**^**a**^	**No**	**Yes**	**No MetS**	**MetS**	**MetS (per 1 unit increase)**
All									
Cases, *N*	746	530	721	1251	1291	706	1337	660	1997
Model 1	1.00	1.07 (0.95 − 1.21)	1.50 (1.33 − 1.69)	1.13 (1.09 − 1.16)	1.00	1.44 (1.30 − 1.60)	1.00	1.41 (1.28 − 1.56)	1.48 (1.37 − 1.61)
Model 2	1.00	1.05 (0.93 − 1.19)	1.44 (1.27 − 1.63)	1.12 (1.09 − 1.16)	1.00	1.39 (1.25 − 1.55)	1.00	1.37 (1.24 − 1.52)	1.42 (1.30 − 1.54)
Men									
Cases, *N*	214	290	528	818	518	514	662	370	1,032
Model 1	1.00	0.94 (0.78 − 1.13)	1.43 (1.20 − 1.69)	1.15 (1.11 − 1.20)	1.00	1.47 (1.29 − 1.68)	1.00	1.51 (1.31 − 1.73)	1.52 (1.36 − 1.70)
Model 2	1.00	0.92 (0.76 − 1.12)	1.32 (1.11 − 1.58)	1.13 (1.09 − 1.18)	1.00	1.37 (1.20 − 1.57)	1.00	1.37 (1.19 − 1.58)	1.35 (1.19 − 1.54)
Women									
Cases, *N*	532	240	193	433	773	192	675	290	965
Model 1	1.00	1.20 (1.02 − 1.41)	1.49 (1.24 − 1.78)	1.08 (1.03 − 1.14)	1.00	1.39 (1.17 − 1.65)	1.00	1.31 (1.13 − 1.52)	1.31 (1.13 − 1.52)
Model 2	1.00	1.17 (0.99 − 1.39)	1.46 (1.21 − 1.75)	1.09 (1.03 − 1.14)	1.00	1.37 (1.15 − 1.63)	1.00	1.30 (1.12 − 1.52)	1.30 (1.12 − 1.52)

Model 1: Stratified by centre, sex and age at recruitment

Model 2: Model 1 additionally adjusted for smoking status, physical activity, lifetime alcohol pattern (never drinker, former drinker, light or never heavy drinker, periodically or always heavy drinker, unknown), education level, HbA1c, fasting status and Mediterranean diet score

*CI* confidence interval, *FLI* fatty liver index, *HR* hazard ratio, *IDF* International Diabetes Federation, *MAFLD* metabolic dysfunction-associated fatty liver disease, *MetS* metabolic syndrome

^a^MetS as a continuous variable excludes those with FLI < 30. P-interaction by sex for categorical FLI (*p* = 0.287), MAFLD (*p* = 0.892) and categorical MetS (*p* = 0.531)

### Cancer mortality

Table 3 shows associations between NAFLD and MetS indices with cancer mortality. A positive association was found between FLI and the risk of cancer mortality with a HR of 1.09 (95%CI = 1.04 − 1.15) per 10-unit increase. In categorical analyses, individuals with an FLI ≥ 60 had a 32% higher risk of cancer mortality (HR 1.32, 95%CI = 1.09 − 1.60) while those with an arbitrary cut-off of FLI ≥ 80 to identify participants with NAFLD, had a 56% higher risk of cancer mortality (HR 1.56, 95%CI = 1.24 − 1.96), compared to those with an FLI < 30 (Additional file 1: Supplementary Table 1). Having MAFLD was positively associated with cancer mortality with a HR of 1.25 (95% CI = 1.06 − 1.48) compared with not having MAFLD. We did not find evidence for heterogeneity of associations by sex between FLI (p-interaction = 0.741), MAFLD (p-interaction = 0.901), or MetS (p-interaction = 0.286) and cancer mortality. Cancer mortality results by sex for all indices are shown in Table 3. In addition, we found evidence for heterogeneity of associations between FLI and cancer mortality by alcohol intake at baseline (Additional file 1: Supplementary Table 2) with a p-interaction = 0.020.

**Table 3:** HRs (95% CIs) for cancer mortality according to FLI, MAFLD and MetS indices, European Prospective Investigation into Cancer, 1992–2000 (*n* = 15,784)

	**FLI**				**MAFLD**		**MetS IDF 2006**		
	**FLI < 30**	**FLI ≥ 30 and < 60**	**FLI ≥ 60**	**FLI (per each 10-point increase)**^**a**^	**No**	**Yes**	**No MetS**	**MetS**	**Mets (per 1 unit increase)**
All									
Cases, N	344	223	268	491	575	260	609	226	835
Model 1	1.00	1.09 (0.91 − 1.31)	1.36 (1.12 − 1.64)	1.09 (1.04 − 1.15)	1.00	1.28 (1.08 − 1.51)	1.00	1.16 (0.98 − 1.36)	1.16 (1.01 − 1.34)
Model 2	1.00	1.08 (0.90 − 1.30)	1.32 (1.09 − 1.60)	1.09 (1.04 − 1.15)	1.00	1.25 (1.06 − 1.48)	1.00	1.14 (0.97 − 1.35)	1.13 (0.98 − 1.30)
Men									
Cases, N	90	119	192	311	216	185	286	115	401
Model 1	1.00	0.94 (0.70 − 1.26)	1.29 (0.98 − 1.69)	1.12 (1.05 − 1.19)	1.00	1.31 (1.06 − 1.62)	1.00	1.09 (0.87 − 1.37)	1.24 (1.02 − 1.51)
Model 2	1.00	0.94 (0.70 − 1.26)	1.24 (0.94 − 1.64)	1.12 (1.04 − 1.19)	1.00	1.26 (1.01 − 1.57)	1.00	1.04 (0.82 − 1.31)	1.20 (0.97 − 1.50)
Women									
Cases, N	254	104	76	180	359	75	323	111	434
Model 1	1.00	1.22 (0.96 − 1.55)	1.33 (1.01 − 1.76)	1.05 (0.97 − 1.14)	1.00	1.23 (0.94 − 1.61)	1.00	1.23 (0.98 − 1.55)	1.09 (0.89 − 1.33)
Model 2	1.00	1.22 (0.95 − 1.56)	1.32 (1.00 − 1.76)	1.06 (0.98 − 1.15)	1.00	1.22 (0.93 − 1.60)	1.00	1.24 (0.98 − 1.57)	1.06 (0.87 − 1.30)

Model 1: stratified by centre, sex and age at recruitment

Model 2: Model 1 additionally adjusted for smoking status, physical activity, lifetime alcohol pattern (never drinker, former drinker, light or never heavy drinker, periodically or always heavy drinker, unknown), education level, HbA1c, fasting status and Mediterranean diet score

*CI* confidence interval, *FLI* fatty liver index, *HR* hazard ratio, *IDF* International Diabetes Federation, *MAFLD* metabolic dysfunction-associated fatty liver disease, *MetS* metabolic syndrome

^a^MetS as a continuous variable excludes those with FLI < 30. P-interaction by sex for categorical FLI (*p* = 0.741), MAFLD (*p* = 0.901) and categorical MetS (*p* = 0.286)

### Cardiovascular disease mortality

Table 4 shows associations between NAFLD and MetS indices with CVD mortality. FLI was positively associated with the risk of CVD mortality, with a HR of 1.15 (95%CI = 1.08 − 1.22) per 10-point increase, and a HR of 2.06 (95%CI = 1.61 − 2.63) for individuals with FLI ≥ 60 compared to FLI < 30. Those with an FLI ≥ 80 displayed a HR of 2.41 (95%CI 1.82 − 3.20) compared to FLI < 30 (Additional file 1: Supplementary Table 1). MAFLD and MetS were positively associated with the risk of CVD mortality, with a HR of 1.84 (95%CI = 1.50 − 2.24) and 1.75 (95%CI = 1.44 − 2.12), respectively. MetS as a continuous variable was also positively associated with CVD mortality with a HR of 1.72 (95%CI = 1.49 − 1.99) per one-unit increase. Associations by sex followed a similar pattern. We also found evidence for heterogeneity of associations with CVD mortality by alcohol intake at baseline for FLI (p-interaction = 0.003; Additional file 1: Supplementary Table 2). As for MetS components, abnormal glucose metabolism was the strongest driver of higher CVD mortality risk (Additional file 1: Supplementary Table 3).

**Table 4:** HRs (95% CIs) for cardiovascular disease mortality according to FLI, MAFLD and MetS indices, European Prospective Investigation into Cancer, 1992–2000 (*n* = 15,784)

	**FLI**				**MAFLD**		**MetS IDF 2006**		
	**FLI < 30**	**FLI ≥ 30 and < 60**	**FLI ≥ 60**	**FLI (per each 10-point increase)**^1^	**No**	**Yes**	**No MetS**	**MetS**	**Mets (per 1 unit increase)**
All									
Cases, N	149	147	224	371	298	222	305	215	520
Model 1	1.00	1.27 (0.99 − 1.63)	2.04 (1.60 − 2.59)	1.15 (1.08 − 1.21)	1.00	1.80 (1.48 − 2.19)	1.00	1.77 (1.47 − 2.13)	1.77 (1.53 − 2.04)
Model 2	1.00	1.28 (0.99 − 1.64)	2.06 (1.61 − 2.63)	1.15 (1.08 − 1.22)	1.00	1.84 (1.50 − 2.24)	1.00	1.75 (1.44 − 2.12)	1.72 (1.49 − 1.99)
Men									
Cases, N	41	80	172	252	123	170	153	140	293
Model 1	1.00	1.26 (0.85 − 1.86)	1.83 (1.53–2.19)	1.21 (1.13 − 1.29)	1.00	2.07 (1.62 − 2.65)	1.00	2.27 (1.78 − 2.90)	1.78 (1.47 − 2.16)
Model 2	1.00	1.29 (0.87 − 1.93)	1.64 (1.33 − 2.03)	1.19 (1.11 − 1.28)	1.00	1.93 (1.50 − 2.49)	1.00	2.05 (1.59 − 2.65)	1.55 (1.24 − 1.95)
Women									
Cases, N	108	67	52	119	175	52	152	75	227
Model 1	1.00	1.35 (0.97 − 1.88)	1.66 (1.37 − 2.02)	1.03 (0.93 − 1.14)	1.00	1.47 (1.05 − 2.07)	1.00	1.24 (0.92 − 1.67)	1.75 (1.41 − 2.16)
Model 2	1.00	1.42 (1.01 − 1.99)	1.70 (1.39 − 2.07)	1.04 (0.93 − 1.15)	1.00	1.53 (1.08 − 2.18)	1.00	1.28 (0.93 − 1.75)	1.78 (1.42 − 2.21)

Model 1: stratified by centre, sex and age at recruitment

Model 2: Model 1 additionally adjusted for smoking status, physical activity, alcohol drinking during lifetime, education level, HbA1c, fasting status and Mediterranean diet score

*CI* confidence interval, *FLI* fatty liver index, *HR* hazard ratio, *IDF* International Diabetes Federation, *MAFLD* metabolic dysfunction-associated fatty liver disease, *MetS* metabolic syndrome, *NA *not available

^1^MetS as a continuous variable excludes those with FLI < 30. P-interaction by sex for categorical FLI (*p* = 0.316), MAFLD (*p* = 0.283) and categorical MetS (*p* = 0.018)

### Any other cause of death

Table 5 shows associations between NAFLD and MetS indices with any other cause of death. All NAFLD and MetS indices were positively associated with the risk of any other cause of death. Most associations in men were not statistically significant. As for MetS components, abnormal glucose metabolism appeared to be the strongest driver of the higher risk of mortality due to any other causes (Additional file 1: Supplementary Table 3).

**Table 5:** HRs (95% CIs) for risk of any other cause of death according to FLI, MAFLD and MetS indices, European Prospective Investigation into Cancer, 1992–2000 (*n* = 15,784)

	**FLI**				**MAFLD**		**MetS IDF 2006**		
	**FLI < 30**	**FLI ≥ 30 and < 60**	**FLI ≥ 60**	**FLI (per each 10-point increase)**^1^	**No**	**Yes**	**No MetS**	**MetS**	**Mets (per 1 unit increase)**
All									
Cases, N	253	160	229	389	414	228	423	219	642
Model 1	1.00	0.93 (0.74 − 1.15)	1.33 (1.07 − 1.64)	1.15 (1.09 − 1.22)	1.00	1.37 (1.14 − 1.65)	1.00	1.49 (1.24 − 1.78)	1.63 (1.42 − 1.88)
Model 2	1.00	0.90 (0.72 − 1.12)	1.21 (0.97 − 1.51)	1.14 (1.08 − 1.21)	1.00	1.27 (1.06 − 1.54)	1.00	1.39 (1.16 − 1.67)	1. 52 (1.32 − 1.75)
Men									
Cases, N	83	91	164	338	175	163	223	115	338
Model 1	1.00	0.77 (0.56 − 1.06)	1.10 (0.83 − 1.47)	1.06 (1.01 − 1.10)	1.00	1.27 (1.01 − 1.60)	1.00	1.49 (1.16 − 1.92)	1.60 (1.32 − 1.93)
Model 2	1.00	0.74 (0.53 − 1.02)	0.96 (0.71 − 1.30)	1.03 (0.99 − 1.08)	1.00	1.15 (0.90 − 1.45)	1.00	1.27 (0.98 − 1.65)	1.37 (1.10 − 1.71)
Women									
Cases, N	170	69	65	110	239	65	200	104	304
Model 1	1.00	1.06 (0.78 − 1.43)	1.59 (1.16 − 2.17)	1.18 (1.07 − 1.32)	1.00	1.56 (1.16 − 2.10)	1.00	1.49 (1.15 − 1.92)	1.67 (1.36 − 2.06)
Model 2	1.00	1.00 (0.73 − 1.36)	1.49 (1.08 − 2.06)	1.19 (1.06 − 1.33)	1.00	1.49 (1.10 − 2.03)	1.00	1.45 (1.11 − 1.89)	1.63 (1.31 − 2.03)

Model 1: stratified by centre, sex and age at recruitment

Model 2: Model 1 additionally adjusted for smoking status, physical activity, lifetime alcohol pattern (never drinker, former drinker, light or never heavy drinker, periodically or always heavy drinker, unknown), education level, HbA1c, fasting status and Mediterranean diet score.NA: not available

*CI* confidence interval, *FLI* fatty liver index, *HR* hazard ratio, *IDF* International Diabetes Federation, *MAFLD* metabolic dysfunction-associated fatty liver disease, *MetS* metabolic syndrome

^1^MetS as a continuous variable excludes those with FLI < 30. P-interaction by sex for categorical FLI (*p* = 0.297), MAFLD (*p* = 0.378) and categorical MetS (*p* = 0.553)

### MASLD and MetALD

Table 6 shows associations between calculated indices of MASLD and MetALD with mortality. We found a higher risk for all-cause mortality in individuals with MASLD (HR = 1.40, 95%CI = 1.26 − 1.56) compared to those without, but the association became stronger in those with MetALD (HR = 1.59, 95%CI = 1.33 − 1.90). Associations between MASLD and MetALD with cancer, cardiovascular mortality or other causes or death were also increased.

**Table 6:** HRs (95% CIs) for mortality risk according to the new nomenclature of metabolic dysfunction-associated steatotic liver disease (MASLD), European Prospective Investigation into Cancer, 1992–2000 (*n* = 15,733)

	**Calculated Index of MASLD**^**a**^		**Calculated Index of METALD**^**b**^	
	**No**	**Yes**	**No**	**Yes**
All-cause mortality				
Cases, *N*	1276	721	1775	213
Model 1	1.00	1.44 (1.31 − 1.61)	1.00	1.71 (1.45 − 2.02)
Model 2	1.00	1.40 (1.26 − 1.56)	1.00	1.59 (1.33 − 1.90)
Cancer mortality				
Cases, *N*	567	268	748	81
Model 1	1.00	1.30 (1.10 − 1.53)	1.00	1.60 (1.23 − 2.08)
Model 2	1.00	1.27 (1.08 − 1.51)	1.00	1.43 (1.08 − 1.88)
Cardiovascular mortality				
Cases, *N*	182	110	259	33
Model 1	1.00	1.60 (1.22 − 2.10)	1.00	2.35 (1.52 − 3.63)
Model 2	1.00	1.60 (1.20 − 2.11)	1.00	2.82 (1.77 − 4.50)
Other causes of death				
Cases, *N*	413	229	571	68
Model 1	1.00	1.38 (1.15 − 1.65)	1.00	1.59 (1.19 − 2.14)
Model 2	1.00	1.28 (1.06 − 1.55)	1.00	1.30 (0.95 − 1.78)

*MetALD* individuals with MASLD who consume more than the recommended amounts of alcohol per week (> 140 g/week and 210 g/week for females and males)

^a^All participants disregarding alcohol consumption. Calculated index of MASLD based on FLI values

^b^Calculated index of MetALD. Comparison of participants with MASLD and with high alcohol intake: > 140 g/week and 210 g/week for females and males, respectively, vs. those with lower alcohol intake

### Sensitivity and additional analyses

In sensitivity analyses (Additional file 1: Supplementary Table 4), findings for all-cause and cause-specific mortality were not meaningfully altered. When excluding those with the highest tertile of sex-specific alcohol intake or periodically and always heavy drinkers during lifetime, the positive associations with mortality remain among those having FLI ≥ 60. Using the MetS IDF 2009 harmonized and NCEP definitions did not drastically alter the findings (Additional file 1: Supplementary Table 5). As for the overlap in indices, we found a positive association with all-cause mortality for individuals with (1) FLI ≥ 60, MAFLD and MetS (HR 1.43, 95%CI 1.25 − 1.64); (2) FLI ≥ 60, MAFLD and phenotypic NASH (HR 1.59, 95% CI 1.20 − 2.10) and (3) FLI ≥ 60, MAFLD, MetS and phenotypic NASH (HR 1.64, 95% CI 1.27 − 2.11). In additional analyses, having phenotypic NASH was positively associated with all-cause mortality (HR = 1.32, 95%CI = 1.11 − 1.56) and CVD mortality (HR = 1.60, 95%CI = 1.18 − 2.17) (data not shown). Heterogeneity of associations between phenotypic NASH and CVD mortality by smoking status (p-interaction = 0.006) in Additional file 1: Supplementary Table 6. The Kaplan Meier curves for all-cause and cause-specific mortality by NAFLD and MetS are shown in Additional file 1: Supplementary Fig. 1.

## Discussion

In this large study based on a prospective cohort, we observe that NAFLD, MAFLD and MetS are associated with increased risk of mortality, particularly from CVD (NAFLD, MAFLD, Mets) and cancer (NAFLD, MAFLD). Of the individual MetS components, abnormal glucose metabolism was the strongest driver of mortality risk associations. Strong, positive risk associations for all-cause, cancer and CVD mortality were also observed for classifications of MASLD and MetALD, which have been proposed to replace NAFLD terminology [6]. Sensitivity analyses did not meaningfully alter our findings. We also observed an even higher risk of mortality for individuals who had higher baseline alcohol consumption levels with NAFLD (all-cause, cancer and CVD mortality). Overall, although our findings further highlight the health implications of metabolic dysfunction and hepatic steatosis. They also demonstrate the need for further assessment and validation of these scores in different populations and studying the impact of interventions aimed at improving metabolic dysfunction and reducing hepatic fat accumulation.

Existing evidence on NAFLD and mortality risk is inconsistent. Two previous global meta-analyses including population- or hospital-based studies and ranging in size from 173 to 11,613 participants showed no statistically significant associations between NAFLD and risk of all-cause, cancer or CVD mortality [1, 12]. In contrast, another meta-analysis of population-based studies ranging in size from 337 to 318,224 participants showed a positive risk association between NAFLD and all-cause mortality but no association with CVD or cancer mortality [13]. However, this meta-analysis included studies with type 2 diabetes, chronic kidney disease or myocardial infarction patients and NAFLD was diagnosed using either abdominal imaging, liver biopsy or ultrasonography. Liver biopsy is the gold standard of NAFLD or NASH diagnoses, but is expensive, invasive and presents side-effects (e.g. bleeding and infection) [34], being impractical for use in large population-based studies. The development and validation of non-invasive indices to predict NAFLD and NASH could potentially be a more cost-effective strategy to identify high-risk individuals for prevention purposes.

In line with our findings, another cohort study in Korea (*n* = 418,296 deaths) [35] has shown positive risk associations between NAFLD and all-cause, CVD and cancer mortality. Similar associations with all-cause mortality have been observed in two smaller European prospective studies [36, 37]. Another prospective study including data from health insurance records (*n* = 14,991 deaths) assessed mortality risks in Finnish individuals with NAFLD at 4 consecutive timepoints between 2009 and 2013 [38]. The study observed that individuals with NAFLD had significantly higher mortality risk than those without NAFLD [38]. A novel aspect of our study is our choice of a second, but arbitrary, cut-off value of FLI ≥ 80, assuming that those with a very high FLI score have more serious degrees of steatosis, needing to be validated in additional studies. This sub-analysis showed strong risk associations for all-cause, cancer and CVD mortality, among those with FLI ≥ 80 compared to those with FLI < 30, with much higher magnitudes of risk than those observed for a cut-off value of FLI ≥ 60.

Our observations of positive associations between MetS and risk of all-cause and cancer mortality are in line with findings from other prospective cohort studies or primary health care data focused on various populations [17, 19, 20, 37, 39, 40]. Our lack of statistical significance for the association of MetS with cancer mortality may be related to study power. Also in line with our findings, previous literature has shown positive associations between MetS and CVD mortality in retrospective and prospective cohort designs or studies using health care data and focusing on specific populations (i.e. American and Iranian) [23, 41–43].

MAFLD is an index which emphasizes the pathogenic association of fatty liver disease with metabolic dysfunction [27] and it is defined as liver steatosis in combination with metabolic dysfunction [9]. MetS plays a major role in driving the perturbations of cardiometabolic homeostasis and its incidence and prevalence positively correlates with that of MAFLD [44]. A recent meta-analysis reported a higher risk of all-cause, cancer and CVD mortality with presence of MAFLD [45], in line with our findings. As for NASH, previous evidence on risk of mortality is scarce and inconsistent [46, 47].

In our study we also compared associations between each individual component of MetS and risk of mortality. We found positive associations between each MetS component with all-cause and CVD mortality, while a higher risk of cancer mortality was observed only for higher abdominal obesity (i.e. ≥ 94 cm in men, ≥ 80 cm in women). Previous evidence on associations between individual MetS components and mortality is inconsistent, with potential reported differences by sex [43], age [48] and ethnicity [49]. A recent meta-analysis of 13 observational cohort studies demonstrated a strong positive association between MetS, but not its individual components, and all-cause mortality [50], whereas an earlier meta-analysis of 20 cohort studies of elderly adults also reported positive all-cause mortality risk associations for higher blood glucose and lower HDL cholesterol, in addition to a positive association for MetS itself [48].

As the different indices each aim to identify some form of metabolic dysfunction, we expected an overlap of findings between them. We found positive risk associations between each index and risk of all-cause mortality. According to our observations, having two or more indices which identify metabolic dysfunction increased the risk of all-cause mortality more than the risk predicted by one single index, thus the duration of metabolic dysfunction and fatty liver disease may have worse health implications. Publications on the duration of metabolic dysfunction and fatty liver disease are only recently appearing and more research is required.

Fatty liver disease nomenclature has recently been reclassified with the term MASLD chosen by consensus to replace NAFLD, with some alterations in the diagnostic definition [6]. Using calculated indices based on FLI to approximate this newer classification, we observe a higher risk of all-cause, cancer and CVD mortality for individuals with MASLD or MetALD compared to those without. Additional studies are needed to validate the FLI against MASLD and MetALD terminology.

The strengths of our study include its prospective design, long follow-up and large sample size, allowing linking of information collected at baseline to mortality at a later point in time. We performed sensitivity analyses to address potential reverse causation by exclusion of the first 2 years of follow-up. We also investigated residual confounding due to chronic inflammation by adjusting for circulating hs-CRP levels and excluding participants with higher alcohol intake levels at baseline, as well as periodically and always heavy drinkers during lifetime. We also looked at heterogeneity by tobacco smoking and excessive alcohol consumption, given their relevance as avoidable causes of death. However, additional studies are required to assess these interactions in different populations. Our study also had limitations. First, only the FLI, MAFLD and MetS indices have been previously validated in different populations, while the phenotypic NASH index still needs validation. Moreover, we utilized FLI-based estimation of hepatic steatosis to calculate indices of MASLD and MetALD. Secondly, we were not able to control for medication use due to data unavailability in our cohort. Another limitation is some misclassification of lifestyle factors. For instance, the FLI does not adequately assess lean NAFLD (i.e. NAFLD in non-obese subjects). Pathophysiological mechanisms justifying the non-obese and lean phenotypes are not well understood, but they may include a more dysfunctional fat, altered body composition, genetic predisposition, epigenetic alterations and different gut microbiota profiles [51]. Fourthly, in our dataset, a large percentage of participants were either not fasting (< 3 h since last meal, 40%) or were in between meals (3–6 h, 16%) at blood collection whereas 14% of participants had missing data. The inability to focus exclusively on fasting subjects may have introduced potential bias in the observed risk associations. Finally, even though we showed higher mortality risk estimates with a cut-point of FLI ≥ 80 in an attempt to identify individuals with a stronger presence of metabolic risk factors, our choice of cut-off was arbitrary and needs to be extensively validated by further studies.

## Conclusions

We conclude that FLI and MetS, non-invasive indices of metabolic dysfunction, are strongly associated with increased risk of all-cause and cause-specific mortality. Broadly similar observations were made for MAFLD. These indices are easier to assess across larger studies than currently used imaging and liver biopsy techniques for assessment of hepatic steatosis. Assessment of the up-to-date classifications of MASLD and MetALD [6] show that both are strongly associated with increased risk of all-cause, cancer and CVD mortality. Additional validation of these indices and their application in studies may facilitate their use for more research, as well as for population-wide screening, surveillance and early diagnosis.

### Supplementary Information

**Additional file 1:** **Supplementary Table 1.** “HRs (95% CIs) for mortality risk according to categorical FLI with an arbitrary cut off of FLI ≥80 to identify participants with NAFLD, European Prospective investigation Into Cancer, 1992-2000 (*n*=15,784)”. **Supplementary Table 2.** “HRs (95% CIs) mortality risk according to FLI categories and sex-specific tertiles of alcohol intake at baseline, European prospective Investigation into Cancer, 1992-2000 (*n*=15,732)”. **Supplementary Table 3.** “HRs (95% CIs) for mortality according to MetS components, European Prospective Investigation into Cancer, 1992-2000 (*n*=15,784)”. **Supplementary Table 4.** “HRs (95% CIs) mortality risk according to FLI and MetS categories in sensitivity analyses, European Prospective Investigation into Cancer, 1992-2000”. **Supplementary Table 5.** “HRs (95% CIs) for mortality risk according to MetS IDF 2009 harmonized and NCEP definition, European Prospective Investigation into Cancer, 1992-2000 (*n*=15,784)”. **Supplementary Table 6.** “HRs (95% CIs) mortality risk according to phenotypic NASH categories and smoking interaction, European prospective Investigation into Cancer, 1992-2000 (*n*=15,612)”. **Supplementary Figure 1.** “Kaplan Meier plots for all-cause and cause specific mortality by liver index”.

## Data Availability

For information on how to apply for access to EPIC data and/or biospecimens, please follow the instructions at http://epic.iarc.fr/access/index.php.

## Abbreviations

ALAT: Alanine aminotransferase
ASAT: Aspartate aminotransferase
BP: Blood pressure
BMI: Body mass index
CVD: Cardiovascular diseases
CI: Confidence interval
CRP: C-reactive protein
EPIC: European Prospective Investigation into Cancer and Nutrition
FPG: Fasting plasma glucose
FLI: Fatty liver index
GGT: Gamma-Glutamyl-Transferase
HRs: Hazard ratios
HCC: Hepatocellular carcinoma
HDL: High-density lipoprotein cholesterol
HOMA-IR: Homeostatic model assessment of insulin resistance
IDF: International Diabetes Federation
MAFLD: Metabolic dysfunction-associated fatty liver disease
MASLD: Metabolic dysfunction-associated steatotic liver disease
MetALD: Metabolic and alcohol-related liver disease
MetS: Metabolic syndrome
MICE: Multivariate imputation by chained equations
NCEP: National Cholesterol Education Program
NAFLD: Non-alcoholic fatty liver disease
NASH: Non-alcoholic steatohepatitis
SD: Standard deviation
TG: Triglycerides
WC: Waist circumference
