# Supplementary material for: Hepatic steatosis, metabolic dysfunction and risk of mortality: findings from a multinational prospective cohort study
Source: BMC Med. 2024 Jun 3;22:221. doi: 10.1186/s12916-024-03366-3 (PMC11145823; doi:10.1186/s12916-024-03366-3)
Supplement: Supplementary file 1 — Additional file 1: Supplementary Table 1. “HRs (95% CIs) for mortality risk according to categorical FLI with an arbitrary cut off of FLI ≥80 to identify participants with NAFLD, European Prospective investigation Into Cancer, 1992-2000 (n=15,784)”. Supplementary Table 2. “HRs (95% CIs) mortality risk according to FLI categories and sex-specific tertiles of alcohol intake at baseline, European prospective Investigation into Cancer, 1992-2000 (n=15,732)”. Supplementary Table 3. “HRs (95% CIs) for mortality according to MetS components, European Prospective Investigation into Cancer, 1992-2000 (n=15,784)”. Supplementary Table 4. “HRs (95% CIs) mortality risk according to FLI and MetS categories in sensitivity analyses, European Prospective Investigation into Cancer, 1992-2000”. Supplementary Table 5. “HRs (95% CIs) for mortality risk according to MetS IDF 2009 harmonized and NCEP definition, European Prospective Investigation into Cancer, 1992-2000 (n=15,784)”. Supplementary Table 6. “HRs (95% CIs) mortality risk according to phenotypic NASH categories and smoking interaction, European prospective Investigation into Cancer, 1992-2000 (n=15,612)”. Supplementary Figure 1. “Kaplan Meier plots for all-cause and cause specific mortality by liver index”. [file 12916_2024_3366_MOESM1_ESM.docx]

**Hepatic Steatosis, Metabolic Dysfunction, and Risk of Mortality: Findings from a Multinational Prospective Cohort Study**

**Supplementary Table 1.** HRs (95% CIs) for mortality risk according to categorical FLI with an arbitrary cut off of FLI ≥80 to identify participants with NAFLD, European Prospective investigation Into Cancer, 1992-2000 (n=15,784).

|  | **FLI** | | | | |  |
| --- | --- | --- | --- | --- | --- | --- |
|  | **FLI<30** | **FLI ≥30 and ≤60** | **FLI≥60 and<80** | **FLI ≥80** | |  |
| *All-cause mortality* |  |  |  |  | |  |
| Cases, N | 746 | 530 | 341 | 380 | |  |
| Model 1 | 1.00 | 1.07 (0.95-1.21) | 1.25 (1.08-1.45) | | 1.81 (1.57-2.09) | |
| Model 2 | 1.00 | 1.05 (0.93-1.19) | 1.21 (1.04-1.40) | | 1.74 (1.50-2.01) | |
|  |  |  |  | |  | |
| *Cancer mortality* |  |  |  | |  | |
| Cases, N | 344 | 223 | 130 | | 138 | |
| Model 1 | 1.00 | 1.09 (0.91-1.31) | 1.18 (0.94-1.48) | | 1.58 (1.26-1.98) | |
| Model 2 | 1.00 | 1.08 (0.89-1.30) | 1.14 (0.90-1.44) | | 1.56 (1.24-1.96) | |
|  |  |  |  | |  | |
| *Cardiovascular disease mortality* | |  |  |  | |  |
| Cases, N | 149 | 147 | 106 | | 118 | |
| Model 1 | 1.00 | 1.27 (0.99-1.63) | 1.75 (1.32-2.31) | 2.40 (1.83-3.16) | |  |
| Model 2 | 1.00 | 1.28 (0.99-1.65) | 1.78 (1.34-2.36) | 2.41 (1.82-3.20) | |  |
|  |  |  |  |  | |  |
| *Any other cause of death* |  |  |  |  | |  |
| Cases, N | 188 | 121 | 80 | | 91 | |
| Model 1 | 1.00 | 0.92 (0.74-1.15) | 1.04 (0.80-1.34) | 1.73 (1.35-2.21) | |  |
| Model 2 | 1.00 | 0.87 (0.68-1.11) | 0.96 (0.71-1.28) | 1.69 (1.28-2.23) | |  |

CI: confidence interval; FLI: fatty liver index; HR: hazard ratio.

Model 1: stratified by centre, sex and age at recruitment.

Model 2: Model 1 additionally adjusted for smoking status, physical activity, lifetime alcohol pattern (never drinker, former drinker, light or never heavy drinker, periodically or always heavy drinker, unknown), education level, HbA1c, fasting status and Mediterranean diet score.

**Supplementary Table 2.** HRs (95% CIs) mortality risk according to FLI categories and sex-specific tertiles of alcohol intake at baseline, European prospective Investigation into Cancer, 1992-2000 (n=15,732).

| **FLI index** | **Alcohol drinking** | **Cases** | **Model 1** | **Model 2** |  |
| --- | --- | --- | --- | --- | --- |
| *All-cause mortality* |  |  |  |  |  |
| FLI<30 | Light | 229 | 1.00 | 1.00 |  |
| FLI<30 | Moderate | 282 | 1.10 (0.91-1.33) | 1.19 (0.98-1.45) |  |
| FLI<30 | Heavy | 232 | 1.08 (0.89-1.32) | 1.14 (0.93-1.41) | |
| FLI ≥30 and <60 | Light | 236 | 1.36 (1.12-1.65) | 1.38 (1.13-1.68) |  |
| FLI ≥30 and <60 | Moderate | 152 | 0.91 (0.73-1.14) | 0.98 (0.78-1.22) |  |
| FLI ≥30 and <60 | Heavy | 140 | 1.13 (0.90-1.42) | 1.12 (0.88-1.42) |  |
| FLI ≥60 | Light | 264 | 1.61 (1.32-1.95) | 1.59 (1.30-1.93) |  |
| FLI ≥60 | Moderate | 202 | 1.36 (1.11-1.67) | 1.45 (1.17-1.79) |  |
| FLI ≥60 | Heavy | 251 | 1.83 (1.49-2.23) | 1.77 (1.43-2.19) |  |
| p-interaction |  |  |  | 0.003 |  |
| *Cancer mortality* | |  |  |  |  |
| FLI <30 | Light | 88 | 1.00 | 1.00 |  |
| FLI <30 | Moderate | 134 | 1.34 (1.00-1.78) | 1.38 (1.03-1.86) |  |
| FLI <30 | Heavy | 121 | 1.28 (0.95-1.72) | 1.26 (0.92-1.72) |  |
| FLI ≥30 and <60 | Light | 94 | 1.58 (1.16-2.16) | 1.62 (1.19-2.21) |  |
| FLI ≥30 and <60 | Moderate | 65 | 1.07 (0.76-1.50) | 1.12 (0.79-1.59) |  |
| FLI ≥30 and <60 | Heavy | 63 | 1.30 (0.92-1.83) | 1.17 (0.82-1.69) |  |
| FLI ≥60 | Light | 85 | 1.41 (1.02-1.95) | 1.41 (1.02-1.97) |  |
| FLI ≥60 | Moderate | 86 | 1.63 (1.18-2.24) | 1.70 (1.22-2.37) |  |
| FLI ≥60 | Heavy | 93 | 1.84 (1.33-2.54) | 1.68 (1.20-2.37) |  |
| p-interaction |  |  |  | 0.020 |  |
| *Cardiovascular disease mortality* | | | |  |  |
| FLI <30 | Light | 46 | 1.00 | 1.00 |  |
| FLI <30 | Moderate | 61 | 1.20 (0.80-1.80) | 1.43 (0.94-2.17) |  |
| FLI <30 | Heavy | 42 | 1.09 (0.70-1.69) | 1.31 (0.82-2.09) |  |
| FLI ≥30 and <60 | Light | 76 | 1.91 (1.29-2.82) | 2.05 (1.38-3.04) |  |
| FLI ≥30 and <60 | Moderate | 44 | 1.07 (0.69-1.66) | 1.19 (0.76-1.89) |  |
| FLI ≥30 and <60 | Heavy | 27 | 1.14 (0.69-1.87) | 1.36 (0.81-2.29) |  |
| FLI ≥60 | Light | 90 | 2.41 (1.64-3.54) | 2.49 (1.68-3.69) |  |
| FLI ≥60 | Moderate | 57 | 1.69 (1.12-2.56) | 1.96 (1.27-3.01) |  |
| FLI ≥60 | Heavy | 77 | 2.76 (1.84-4.14) | 3.48 (2.26-5.35) |  |
| p-interaction |  |  |  | 0.003 |  |
| *Any other cause of death* | |  |  |  |  |
| FLI <30 | Light | 95 | 1.00 | 1.00 |  |
| FLI <30 | Moderate | 87 | 0.83 (0.60-1.14) | 0.91 (0.65-1.27) |  |
| FLI <30 | Heavy | 69 | 0.88 (0.63-1.24) | 0.93 (0.65-1.34) |  |
| FLI ≥30 and <60 | Light | 66 | 0.88 (0.63-1.23) | 0.86 (0.61-1.22) |  |
| FLI ≥30 and <60 | Moderate | 43 | 0.69 (0.47-1.03) | 0.75 (0.50-1.12) |  |
| FLI ≥30 and <60 | Heavy | 50 | 0.97 (0.67-1.41) | 0.95 (0.64-1.41) |  |
| FLI ≥60 | Light | 89 | 1.34 (0.97-1.84) | 1.28 (0.92-1.78) |  |
| FLI ≥60 | Moderate | 59 | 0.96 (0.67-1.37) | 1.00 (0.69-1.45) |  |
| FLI ≥60 | Heavy | 81 | 1.35 (0.96 - 1.9) | 1.19 (0.82-1.72) |  |
| p-interaction |  |  |  | 0.895 |  |

CI: confidence interval; FLI: fatty liver index; HR: hazard ratio.

Model 1: Stratified by centre sex and age at recruitment.

Model 2: Model 1 additionally adjusted for smoking status, physical activity, lifetime alcohol pattern (never drinker, former drinker, light or never heavy drinker, periodically or always heavy drinker, unknown), education level, HbA1c, fasting status and Mediterranean diet score.

**Supplementary Table 3.** HRs (95% CIs) for mortality according to MetS components, European Prospective Investigation into Cancer, 1992-2000 (n=15,784).

| **MetS components** | **Model 1** | **Model 2** |
| --- | --- | --- |
| *All-cause mortality* |  |  |
| Abnormal glucose metabolism | 1.49 (1.30-1.71) | 1.41 (1.22-1.61) |
| Elevated TG | 1.19 (1.08-1.32) | 1.14 (1.03-1.26) |
| Elevated BP | 1.19 (1.08-1.31) | 1.18 (1.07-1.30) |
| Abdominal obesity (IDF definition) | 1.31 (1.18-1.44) | 1.29 (1.17-1.43) |
| Reduced HDL cholesterol | 1.44 (1.24-1.68) | 1.31 (1.12-1.52) |
| BMI (per 1kg/m^2^ increase) | 1.02 (1.01-1.04) | 1.02 (1.01-1.04) |
| *Cancer mortality* |  |  |
| Abnormal glucose metabolism | 1.00 (0.78-1.28) | 0.96 (0.75-1.23) |
| Elevated TG (corrected by fasting status) | 1.14 (0.98-1.33) | 1.10 (0.94-1.29) |
| Elevated BP | 0.86 (0.73-1.00) | 0.86 (0.74-1.01) |
| Abdominal obesity (IDF definition) | 1.26 (1.09-1.47) | 1.25 (1.07-1.46) |
| Reduced HDL cholesterol | 1.24 (0.97-1.57) | 1.14 (0.89-1.46) |
| BMI (per 1kg/m^2^ increase) | 1.01 (0.99-1.03) | 1.02 (1.00-1.03) |
| *Cardiovascular disease mortality* | |  |
| Abnormal glucose metabolism | 2.14 (1.70-2.71) | 2.06 (1.63-2.61) |
| Elevated TG (corrected by fasting status) | 1.39 (1.16-1.68) | 1.32 (1.09-1.59) |
| Elevated BP | 1.63 (1.35-1.97) | 1.61 (1.33-1.94) |
| Abdominal obesity (IDF definition) | 1.60 (1.31-1.95) | 1.62 (1.33-1.99) |
| Reduced HDL cholesterol | 1.83 (1.39-2.41) | 1.68 (1.27-2.21) |
| BMI (per 1kg/m^2^ increase) | 1.05 (1.02-1.07) | 1.05 (1.03-1.08) |
| *Any other cause of death* |  |  |
| Abnormal glucose metabolism | 1.62 (1.27- 2.06) | 1.49 (1.16-1.90) |
| Elevated TG (corrected by fasting status) | 1.10 (0.92-1.31) | 1.03 (0.86-1.23) |
| Elevated BP | 1.38 (1.17-1.64) | 1.32 (1.11-1.57) |
| Abdominal obesity (IDF definition) | 1.16 (0.98-1.39) | 1.12 (0.94-1.34) |
| Reduced HDL cholesterol | 1.42 (1.09-1.87) | 1.27 (0.96-1.68) |
| BMI (per 1kg/m^2^ increase) | 1.02 (1.00-1.04) | 1.01 (1.00-1.04) |

BMI: body mass index; BP: blood pressure; CI: confidence interval; HDL: high-density lipoprotein; HR: hazard ratio; IDF: International Diabetes Federation; MetS: metabolic syndrome; TG: triglycerides. Abnormal glucose metabolism (raised fasting plasma glucose (FPG) ≥100 mg/dL or previously diagnosed type 2 diabetes), elevated TG (≥150 mg/dL or specific treatment for this lipid abnormality), elevated BP (systolic BP ≥130 mmHg, diastolic BP ≥ 85 mmHg or treatment of previously diagnosed hypertension), abdominal obesity (WC >94 cm in men or >80 cm in women), reduced HDL cholesterol (<40 mg/dL in men, <50 mg/dL in women).

Model 1: Stratified by centre, sex and age at recruitment.

Model 2: Model 1 additionally adjusted for smoking status, physical activity, lifetime alcohol pattern (never drinker, former drinker, light or never heavy drinker, periodically or always heavy drinker, unknown), education level, HbA1c, fasting status and Mediterranean diet score.

**Supplementary Table 4.** HRs (95% CIs) mortality risk according to FLI and MetS categories in sensitivity analyses, European Prospective Investigation into Cancer, 1992-2000.

|  |  | **FLI** | | |  |  | **MAFLD** | |  | **Phenotypic NASH** | |  | **MetS IDF 2006** | | |
| --- | --- | --- | --- | --- | --- | --- | --- | --- | --- | --- | --- | --- | --- | --- | --- |
|  | **Cases** | **FLI<30** | **FLI ≥30**  **and <60** | **FLI≥60** | **FLI (per each 10 point increase)** |  | **No** | **Yes** |  | **No NASH** | **NASH** |  | **No MetS** | **MetS** | **MetS (per 1 unit increase)** |
| *All-cause mortality* | | | | |  |  |  | | | | | | | | |
| Model 1 | 1,997 | 1.00 | 1.07 (0.95-1.21) | 1.50 (1.33-1.69) | 1.08 (1.06-1.10) |  | 1.00 | 1.44 (1.30-1.60) |  | 1.00 | 1.29 (1.09-1.53) |  | 1.00 | 1.41 (1.28-1.56) | 1.48 (1.37-1.61) |
| Model 2 | 1,997 | 1.00 | 1.04 (0.92-1.18) | 1.41 (1.25-1.60) | 1.07 (1.05-1.09) |  | 1.00 | 1.37 (1.23-1.52) |  | 1.00 | 1.31 (1.10-1.56) |  | 1.00 | 1.37 (1.23-1.51) | 1.38 (1.28-1.48) |
| Model 3 | 1,918 | 1.00 | 1.03 (0.91-1.17) | 1.45 (1.28-1.64) | 1.07 (1.05-1.09) |  | 1.00 | 1.41 (1.27-1.57) |  | 1.00 | 1.37 (1.15-1.63) |  | 1.00 | 1.38 (1.25-1.54) | 1.40 (1.30-1.51) |
| Model 4 | 1,374 | 1.00 | 1.10 (0.95-1.28) | 1.41 (1.21-1.64) | 1.06 (1.03-1.09) |  | 1.00 | 1.35 (1.19-1.54) |  | 1.00 | 1.13 (0.90-1.42) |  | 1.00 | 1.36 (1.20-1.54) | 1.42 (1.28-1.56) |
| Model 5 | 1,748 | 1.00 | 1.05 (0.92-1.20) | 1.43 (1.25-1.63) | 1.07 (1.05-1.09) |  | 1.00 | 1.39 (1.24-1.56) |  | 1.00 | 1.29 (1.07-1.55) |  | 1.00 | 1.39 (1.24-1.55) | 1.40 (1.28-1.53) |
| Model 6 | 866 | 1.00 | 0.99 (0.82-1.20) | 1.30 (1.07-1.59) | 1.05 (1.02-1.08) |  | 1.00 | 1.27 (1.07- 1.51) |  | 1.00 | 1.26 (0.93-1.70) |  | 1.00 | 1.31 (1.10-1.56) | 1.37 (1.19-1.58) |
| Model 7 | 995 | 1.00 | 0.99 (0.82-1.18) | 1.55 (1.29-1.85) | 1.08 (1.05–1.10) |  | 1.00 | 1.54 (1.32-1.79) |  | 1.00 | 1.37 (1.06-1.76) |  | 1.00 | 1.57 (1.36-1.81) | 1.66 (1.49-1.86) |
| Model 8 | 1,997 | NA^1^ | NA | NA | NA |  | NA | NA |  | NA | NA |  | 1.00 | 1.32 (1.18-1.48) | 1.39 (1.27-1.52) |
| Model 9 | 1,997 | 1.00 | 1.04 (0.92-1.18) | 1.37 (1.21-1.55) | 1.06 (1.04-1.08) |  | NA | NA |  | NA | NA |  | NA | NA | NA |
| *Cancer mortality* | |  |  |  |  |  |  |  |  |  |  |  |  |  |  |
| Model 1 | 835 | 1.00 | 1.09 (0.91-1.31) | 1.36 (1.12-1.64) | 1.05 (1.02-1.08) |  | 1.00 | 1.28 (1.08-1.51) |  | 1.00 | 1.04 (0.78-1.39) |  | 1.00 | 1.16 (0.98-1.36) | 1.16 (1.01-1.34) |
| Model 2 | 835 | 1.00 | 1.07 (0.89-1.30) | 1.31 (1.08-1.58) | 1.05 (1.02-1.08) |  | 1.00 | 1.24 (1.04-1.46) |  | 1.00 | 1.07 (0.80-1.44) |  | 1.00 | 1.37 (1.23-1.51) | 1.13 (0.99-1.28) |
| Model 3 | 805 | 1.00 | 1.06 (0.88-1.29) | 1.30 (1.07-1.58) | 1.05 (1.02-1.08) |  | 1.00 | 1.24 (1.04-1.47) |  | 1.00 | 1.05 (0.78-1.42) |  | 1.00 | 1.15 (0.97-1.36) | 1.15 (1.01-1.30) |
| Model 4 | 558 | 1.00 | 1.21 (0.93-1.57) | 1.36 (1.08-1.72) | 1.05 (1.01-1.09) |  | 1.00 | 1.24 (1.01-1.53) |  | 1.00 | 0.87 (0.59-1.27) |  | 1.00 | 1.11 (0.91-1.37) | 1.16 (0.98-1.38) |
| Model 5 | 724 | 1.00 | 1.07 (0.88-1.31) | 1.26 (1.03-1.55) | 1.04 (1.01-1.07) |  | 1.00 | 1.23 (1.02-1.48) |  | 1.00 | 0.99 (0.72-1.36) |  | 1.00 | 1.15 (0.96-1.37) | 1.12 (0.96-1.30) |
| Model 6 | 393 | 1.00 | 0.98 (0.74-1.30) | 1.15 (0.85-1.55) | 1.02 (0.97-1.07) |  | 1.00 | 1.11 (0.85-1.45) |  | 1.00 | 0.96 (0.59-1.57) |  | 1.00 | 1.05 (0.80-1.39) | 1.05 (0.84-1.31) |
| Model 7 | 80 | 1.00 | 0.87 (0.46-1.63) | 0.77 (0.35-1.72) | 1.00 (0.89-1.12) |  | 1.10 | 0.78 (0.38-1.60) |  | 1.00 | 0.50 (0.14-1.85) |  | 1.00 | 1.44 (0.79-2.65) | 1.73 (0.96-3.1) |
| Model 8 | 835 | NA | NA | NA | NA |  | NA | NA |  | NA | NA |  | NA | NA | NA |
| Model 9 | 835 | 1.00 | 1.08 (0.89-1.30) | 1.31 (1.08-1.59) | 1.05 (1.02-1.08) |  | NA | NA |  | NA | NA |  | NA | NA | NA |
| *Cardiovascular disease mortality* | | | |  |  |  |  |  |  |  |  |  |  |  |  |
| Model 1 | 520 | 1.00 | 1.27 (0.99-1.63) | 2.04 (1.60-2.59) | 1.12 (1.09-1.16) |  | 1.00 | 1.80 (1.48-2.19) |  | 1.00 | 1.42 (1.06- 1.91) |  | 1.00 | 1.77 (1.47-2.13) | 1.77 (1.53-2.04) |
| Model 2 | 520 | 1.00 | 1.26 (0.98-1.63) | 2.02 (1.58-2.58) | 1.12 (1.08-1.16) |  | 1.00 | 1.78 (1.46-2.18) |  | 1.00 | 1.49 (1.10-2.02) |  | 1.00 | 1.74 (1.44-2.10) | 1.70 (1.47-1.97) |
| Model 3 | 490 | 1.00 | 1.23 (0.94-1.59) | 2.05 (1.59-2.63) | 1.13 (1.09-1.17) |  | 1.00 | 1.84 (1.50-2.24) |  | 1.00 | 1.62 (1.20- 2.19) |  | 1.00 | 1.75 (1.44-2.13) | 1.74 (1.49-2.02) |
| Model 4 | 374 | 1.00 | 1.34 (0.99-1.81) | 1.84 (1.37-2.48) | 1.10 (1.05-1.15) |  | 1.00 | 1.56 (1.23-1.99) |  | 1.00 | 1.15 (0.76-1.72) |  | 1.00 | 1.69 (1.34-2.12) | 1.66 (1.40-1.96) |
| Model 5 | 479 | 1.00 | 1.30 (1.00-1.68) | 1.96 (1.52-2.52) | 1.12 (1.08-1.16) |  | 1.00 | 1.71 (1.38–2.10) |  | 1.00 | 1.45 (1.05–2.01) |  | 1.00 | 1.71 (1.40- 2.10) | 1.68 (1.44-1.96) |
| Model 6 | 157 | 1.00 | 1.28 (0.77-2.12) | 2.49 (1.54-4.02) | 1.15 (1.07-1.24) |  | 1.00 | 2.15 (1.46-3.14) |  | 1.00 | 1.63 (0.86-3.10) |  | 1.00 | 1.71 (1.16-2.51) | 1.70 (1.28-2.25) |
| Model 7 | 424 | 1.00 | 1.24 (0.93-1.64) | 2.14 (1.62-2.84) | 1.14 (1.09-1.18) |  | 1.00 | 1.93 (1.54-2.43) |  | 1.00 | 1.48 (1.04-2.12) |  | 1.00 | 1.76 (1.42–2.19) | 1.78 (1.52-2.09) |
| Model 8 | 520 | NA | NA | NA | NA |  | NA | NA |  | NA | NA |  | 1.00 | 1.74 (1.44-2.11) | 1.72 (1.49-1.99) |
| Model 9 | 520 | 1.00 | 1.27 (1.00-1.64) | 1.91 (1.50-2.46) | 1.11 (1.07-1.15) |  | NA | NA |  | NA | NA |  | NA | NA | NA |
| *Any other cause of death* | | | | |  |  |  |  |  |  |  |  |  |  |  |
| Model 1 | 642 | 1.00 | 0.93 (0.74-1.15) | 1.33 (1.07-1.64) | 1.07 (1.03-1.10) |  | 1.00 | 1.37 (1.14-1.65) |  | 1.00 | 1.53 (1.14-2.05) |  | 1.00 | 1.49 (1.24-1.78) | 1.63 (1.42-1.88) |
| Model 2 | 642 | 1.00 | 0.88 (0.71-1.10) | 1.19 (0.95-1.47) | 1.05 (1.01-1.08) |  | 1.00 | 1.26 (1.04-1.52) |  | 1.00 | 1.45 (1.07-1.95) |  | 1.00 | 1.38 (1.15-1.66) | 1.5 (1.30-1.73) |
| Model 3 | 623 | 1.00 | 0.90 (0.72-1.13) | 1.27 (1.02-1.58) | 1.06 (1.03-1.10) |  | 1.00 | 1.33 (1.10-1.61) |  | 1.00 | 1.53 (1.13- 2.06) |  | 1.00 | 1.42 (1.18-1.71) | 1.53 (1.32-1.77) |
| Model 4 | 442 | 1.00 | 0.87 (0.66-1.14) | 1.18 (0.91-1.54) | 1.05 (1.01-1.09) |  | 1.00 | 1.25 (0.99-1.58) |  | 1.00 | 1.46 (1.01-2.11) |  | 1.00 | 1.42 (1.13-1.77) | 1.51 (1.27-1.79) |
| Model 5 | 545 | 1.00 | 0.87 (0.69-1.11) | 1.26 (0.99-1.59) | 1.06 (1.02-1.10) |  | 1.00 | 1.34 (1.09-1.64) |  | 1.00 | 1.53 (1.10-2.13) |  | 1.00 | 1.45 (1.19-1.78) | 1.51 (1.29-1.77) |
| Model 6 | 316 | 1.00 | 0.96 (0.69-1.32) | 1.11 (0.79–1.55) | 1.04 (0.99-1.10) |  | 1.00 | 1.12 (0.84-1.49) |  | 1.00 | 1.40 (0.86-2.28) |  | 1.00 | 1.41 (1.05-1.89) | 1.58 (1.25-1.99) |
| Model 7 | 491 | 1.00 | 0.85 (0.65-1.10) | 1.31 (1.02-1.69) | 1.06 (1.02-1.10) |  | 1.00 | 1.37 (1.10-1.71) |  | 1.00 | 1.43 (0.98-2.11) |  | 1.00 | 1.41 (1.14-1.75) | 1.57 (1.33-1.85) |
| Model 8 | 642 | 1.00 | NA | NA | NA |  | NA | NA |  | NA | NA |  | NA | 1.39 (1.14-1.70) | 1.54 (1.32-1.80)) |
| Model 9 | 642 | 1.00 | 0.88 (0.69-1.13) | 1.18 (0.87-1.60) | NA |  | NA | NA |  | NA | NA |  | NA | NA | NA |

CI: confidence interval; FLI: fatty liver index; HR: hazard ratio; IDF: International Diabetes Federation; MAFLD: metabolic dysfunction-associated fatty liver disease; MetS: metabolic syndrome; NASH: non-alcoholic steatohepatitis.

^1^ NA stands for not applicable whenever it was not possible to adjust for the indicated variable as it is part of the formula to construct the referred index.

Model 1: Stratified by centre, sex and age at recruitment.

Model 2: adjusted for smoking status, physical activity, alcohol intake during lifetime, schooling, HbA1c, fasting status, Mediterranean diet score and hs-CRP.

Model 3: adjusted for smoking status, physical activity, alcohol intake during lifetime, schooling, HbA1c, fasting status, Mediterranean diet and excluding first 2 yrs of follow-up.

Model 4: adjusted for smoking status, physical activity, alcohol intake during lifetime, schooling, HbA1c, fasting status, Mediterranean diet score excluding those with the highest tertile of sex-specific alcohol intake (26 to 276 g/day of alcohol in men, 8 to 143g/day of alcohol in women).

Model 5: adjusted for smoking status, physical activity, alcohol intake during lifetime, schooling, HbA1c, fasting status, Mediterranean diet score excluding periodically and always heavy drinkers during lifetime.

Model 6: adjusted for smoking status, physical activity, alcohol intake during lifetime, schooling, HbA1c, fasting status, Mediterranean diet score excluding individuals who reported a cardiovascular problem at baseline.

Model 7: adjusted for smoking status, physical activity, alcohol intake during lifetime, schooling, HbA1c, fasting status, Mediterranean diet score excluding individuals who reported any type of incident cancer.

Model 8: adjusted for smoking, physical activity, alcohol intake during lifetime, schooling, HbA1c, fasting status, Mediterranean diet score and BMI.

Model 9: adjusted for smoking, physical activity, alcohol intake during lifetime, schooling, fasting status, Mediterranean diet score and glucose, instead of HbA1c.

**Supplementary Table 5.** HRs (95% CIs) for mortality risk according to MetS IDF 2009 harmonized and NCEP definition, European Prospective Investigation into Cancer, 1992-2000 (n=15,784).

| **MetS** | **IDF 2009** | | **NCEP** | | |  |
| --- | --- | --- | --- | --- | --- | --- |
|  | **No** | **Yes** | **No** | **Yes** | |  |
| *All-cause mortality* |  |  |  |  | |  |
| Cases, N | 1,294 | 703 | 1,540 | 457 | |  |
| Model 1 | 1.00 | 1.43 (1.29-1.58) | 1.00 | | 1.44 (1.29-1.61) | |
| Model 2 | 1.00 | 1.38 (1.25-1.53) | 1.00 | | 1.37 (1.22-1.54) | |
|  |  |  |  | |  | |
| *Cancer mortality* |  |  |  | |  | |
| Cases, N | 593 | 242 | 686 | | 149 | |
| Model 1 | 1.00 | 1.17 (0.99-1.37) | 1.00 | | 1.12 (0.93-1.36) | |
| Model 2 | 1.00 | 1.14 (0.97-1.34) | 1.00 | | 1.09 (0.90-1.32) | |
|  |  |  |  | |  | |
| *Cardiovascular disease mortality* | |  |  |  | |  |
| Cases, N | 294 | 226 | 371 | | 149 | |
| Model 1 | 1.00 | 1.76 (1.46-2.12) | 1.00 | 1.75 (1.42-2.15) | |  |
| Model 2 | 1.00 | 1.73 (1.43-2.09) | 1.00 | 1.67 (1.36-2.06) | |  |
|  |  |  |  |  | |  |
| *Any other cause of death* |  |  |  |  | |  |
| Cases, N | 304 | 176 | 359 | | 121 | |
| Model 1 | 1.00 | 1.48 (1.20-1.83) | 1.00 | 1.69 (1.34-2.13) | |  |
| Model 2 | 1.00 | 1.50 (1.22-1.85) | 1.00 | 1.60 (1.27-2.02) | |  |

CI: confidence interval; HR: hazard ratio; IDF: International Diabetes Federation; MetS: metabolic syndrome; NCEP: National Cholesterol Education Program.

Model 1: stratified by center, sex and age at recruitment.

Model 2: Model 1 additionally adjusted for smoking status, physical activity, lifetime alcohol pattern (never drinker, former drinker, light or never heavy drinker, periodically or always heavy drinker, unknown), education level, HbA1c, fasting status and Mediterranean diet score.

**Supplementary Table 6.** HRs (95% CIs) mortality risk according to phenotypic NASH categories and smoking interaction, European prospective Investigation into Cancer, 1992-2000 (n=15,612).

| **Phenotypic NASH** | **Smoking status** | **Cases** | **Model 1** | **Model 2** |
| --- | --- | --- | --- | --- |
| *All-cause mortality* | |  |  |  |
| No NASH | Never | 590 | 1.00 | 1.00 |
| No NASH | Former | 559 | 1.36 (1.20-1.55) | 1.37 (1.20-1.56) |
| No NASH | Current | 643 | 2.36 (2.08-2.68) | 2.29 (2.02-2.61) |
| NASH | Never | 46 | 1.14 (0.83-1.56) | 1.11 (0.81-1.53) |
| NASH | Former | 70 | 1.98 (1.52-2.59) | 1.91 (1.46-2.49) |
| NASH | Current | 61 | 3.51 (2.61-4.72) | 3.53 (2.62-4.75) |
| p-interaction |  |  |  | 0.489 |
|  |  |  |  |  |
| *Cancer mortality* |  |  |  |  |
| No NASH | Never | 241 | 1.00 | 1.00 |
| No NASH | Former | 227 | 1.39 (1.14-1.70) | 1.40 (1.14-1.71) |
| No NASH | Current | 290 | 2.26 (1.87-2.74) | 2.23 (1.83- 2.71) |
| NASH | Never | 17 | 1.01 (0.60-1.69) | 1.03 (0.62-1.73) |
| NASH | Former | 22 | 1.53 (0.97-2.43) | 1.50 (0.94-2.38) |
| NASH | Current | 24 | 3.13 (1.95-5.01) | 3.09 (1.92-4.96) |
| p-interaction |  |  |  | 0.637 |
|  |  |  |  |  |
| *Cardiovascular disease mortality* | | |  |  |
| No NASH | Never | 157 | 1.00 | 1.00 |
| No NASH | Former | 147 | 1.27 (0.99-1.64) | 1.30 (1.00-1.68) |
| No NASH | Current | 155 | 2.33 (1.81-3.00) | 2.28 (1.76-2.95) |
| NASH | Never | 10 | 0.76 (0.39-1.48) | 0.75 (0.39-1.47) |
| NASH | Former | 23 | 1.97 (1.23-3.16) | 2.01 (1.25-3.24) |
| NASH | Current | 24 | 5.66 (3.45-9.27) | 6.12 (3.70-10.11) |
| p-interaction |  |  |  | 0.006 |
|  |  |  |  |  |
| *Any other cause of death* | |  |  |  |
| No NASH | Never | 192 | 1.00 | 1.00 |
| No NASH | Former | 185 | 1.40 (1.12 - 1.77) | 1.40 (1.11 - 1.77) |
| No NASH | Current | 198 | 2.54 (2.02 - 3.20) | 2.40 (1.89 - 3.04) |
| NASH | Never | 19 | 1.82 (1.09 - 3.03) | 1.70 (1.01 - 2.85) |
| NASH | Former | 25 | 2.69 (1.71 - 4.25) | 2.48 (1.56 - 3.94) |
| NASH | Current | 13 | 2,33 (1.26 - 4.30) | 2.20 (1.18 - 4.11) |
| p-interaction |  |  |  | 0.187 |

CI: confidence interval; HR: hazard ratio; NASH: non-alcoholic steatohepatitis.

Model 1: Stratified by centre, sex and age at recruitment.

Model 2: Model 1 additionally adjusted for physical activity, lifetime alcohol pattern (never drinker, former drinker, light or never heavy drinker, periodically or always heavy drinker, unknown), education level, HbA1c, fasting status and Mediterranean diet score.

Supplementary Figure 1. Kaplan Meier plots for all-cause and cause specific mortality by liver index

a)

b)

d)

c)

1. All-cause mortality (n=1,997; b) cancer mortality (n=835); c) cardiovascular mortality (n=520); d) any other cause of mortality (n=642 deaths).
